# Exercise Increases and Browns Muscle Lipid in High-Fat Diet-Fed Mice

**DOI:** 10.3389/fendo.2016.00080

**Published:** 2016-06-30

**Authors:** Tiffany L. Morton, Kornelia Galior, Cody McGrath, Xin Wu, Gunes Uzer, Guniz Bas Uzer, Buer Sen, Zhihui Xie, David Tyson, Janet Rubin, Maya Styner

**Affiliations:** ^1^Department of Medicine, Division of Endocrinology and Metabolism, University of North Carolina at Chapel Hill, Chapel Hill, NC, USA

**Keywords:** intramyocellular lipid, running, exercise, brown adipose tissue

## Abstract

Muscle lipid increases with high-fat feeding and diabetes. In trained athletes, increased muscle lipid is not associated with insulin resistance, a phenomenon known as the *athlete’s paradox*. To understand if exercise altered the phenotype of muscle lipid, female C57BL/6 mice fed CTL or high-fat diet (HFD for 6 or 18 weeks) were further divided into sedentary or exercising groups (CTL-E or HFD-E) with voluntary access to running wheels for the last 6 weeks of experiments, running 6 h/night. Diet did not affect running time or distance. HFD mice weighed more than CTL after 18 weeks (*p* < 0.01). Quadriceps muscle TG was increased in running animals and in sedentary mice fed HFD for 18 weeks (*p* < 0.05). In exercised animals, markers of fat, *Plin1, aP2, FSP27*, and *Fasn*, were increased significantly in HFD groups. *Ucp1* and *Pgc1a*, markers for brown fat, increased with exercise in the setting of high fat feeding. *Fndc5*, which encodes irisin, and *CytC* were sensitive to exercise regardless of diet. *Plin5* was increased with HFD and unaffected by exercise; the respiratory exchange ratio was 15% lower in the 18-week HFD group compared with CTL (*p* < 0.001) and 10% lower in 18 weeks HFD-E compared with CTL-E (*p* < 0.001). Increased *Ucp1* and *Pgc1a* in exercised muscle of running mice suggests that a beige/brown fat phenotype develops, which differs from the fat phenotype that induces insulin resistance in high fat feeding. This suggests that increased muscle lipid may develop a “brown” phenotype in the setting of endurance exercise training, a shift that is further promoted by HFD.

## Introduction

Accumulation of muscle lipid has been associated with obesity, insulin resistance, and diabetes (1–3). High-fat diet and lipid infusion both increase muscle lipid (4). Diet-induced weight loss has been shown to decrease muscle lipid while also improving insulin sensitivity, further supporting a negative association of muscle fat with metabolic parameters (3). In direct contrast to these negative associations of muscle lipid, muscle of endurance-trained athletes – who are insulin sensitive – also has been shown to harbor increased muscle lipid, a phenomenon referred to as the *athlete’s paradox* (5). The muscle lipid quantity in a trained athlete has been noted to be even greater than that of obese and diabetic subjects (6). The *paradox* of known high muscle lipid in athletes, as well as in diabetic patients, remains poorly understood.

Upon initiation of exercise, there is an increase in uptake and oxidation of lipids in skeletal muscle (7). When exercise intensity increases, fuel selection appears to shift toward an increase in carbohydrate and decrease in fat utilization. By contrast, endurance training is associated with a shift toward an enhanced lipid utilization (7). During short-term maximal exercise, muscle ATP synthesis is principally achieved *via* breakdown of creatine phosphate and during the conversion of glucose units, derived mostly from glycogen. Contribution of carbohydrate fuels increases with rising exercise intensity, simultaneous with a reduction in lipid oxidation. Conversely, during sustained exercise at fixed moderate intensity, carbohydrate oxidation rates decline as fat oxidation rates increase (8). Brown adipose tissue, initially noted in hibernating mammals and human infants, functions to dissipate energy in the form of heat through non-shivering thermogenesis (9). Recently, inducible brown fat depots (beige fat) have been discovered within the white adipose tissue of adult humans (10). On exposure to cold or β-adrenergic stimulation these beige/brite fat cells express high levels of mitochondrial uncoupling protein UCP1 and fat globules become multilocular (11), characteristics of the brown fat phenotype. Irisin, a muscle-derived hormone induced by exercise, also activates UCP1 expression and browning of white adipose tissue (12): co-activator PPAR-γ co-activator-1 α (PGC1-α) stimulates irisin and transgenic mice with overexpression of PGC1α exhibit increased energy expenditure despite no changes in food intake or activity (12). Overall, there is evidence that fat depots can alter phenotype to serve functional demands.

Since exercise browns white adipose depots (12) and sympathomimetics, which stimulate brown adipose tissue formation, both increase muscle lipid (13), we hypothesized that exercise might result in an analogous browning of muscle lipid. As such, the muscle lipid contributing to the *athlete’s paradox* might represent an increase in brown fat. We also hypothesized that increased lipid in the diet through high fat feeding, as seen in humans (14), would potentiate muscle lipid accumulation in exercised muscle, thus increasing the amount of muscle fat susceptible to “browning.”

To test our hypotheses, we studied the effect of running exercise on white and brown adipose tissue markers in skeletal muscle in an exercising rodent model. Mice provided voluntary access to running wheels run for up to 6 h nightly, a level consistent with endurance training (15–17). We were able to demonstrate a phenotypic switch in response to running exercise: markers of brown fat increased in the setting of exercise. Interestingly, high-fat diet (HFD) feeding both short term and long term, causing an overall increase in muscle lipid, significantly augmented our ability to measure exercise-induced browning of muscle lipid.

## Materials and Methods

### Short-term High-Fat Diet

The UNC IACUC approved the use and care of animals. Eight-week-old female C57BL/6 mice (*n* = 20) were assigned to one of two diets for a period of 6 weeks: control diet (PicoLab Mouse Diet 20, Item #5058) or *ad libitum* short-term HFD feeding high-fat diet consisting of 45% calories from fat (#D12451, Research diets). At the beginning of the experiment, mice were further divided into exercisers (with voluntary access to running wheels) or non-exercisers for a 6-week period. Female C57BL/6 mice were used because this gender and strain has been shown to be highly motivated to exercise daily when provided with access to running wheels (16). The groups were as follows: control or CTL (*n* = 5), HFD (*n* = 5), Control Exercise or CTL-E (*n* = 5), and HFD Exercise or HFD-E (*n* = 5).

### Long-term High-Fat Diet

Four-week-old C57BL/6 female mice were fed regular chow diet (*n* = 14) or HFD (#D12451, Research diets) (*n* = 14) beginning at age of 4 weeks. After 12 weeks, control mice were allocated to low-fat diet (#D12450B, Research Diets); the high-fat diet group was maintained on HFD (#D12451, Research diets). The mice were further divided into sedentary (CTL *n* = 7, HFD *n* = 7) or exercise groups (CTL-E *n* = 7, HFD-E *n* = 7). The exercise group was provided access to voluntary running wheels. The chow and LFD diets are marginally different in fat content (13.427% kcal of fat in ctrl; 10% kcal of fat in LFD). Both experiments (short term and long term) are analyzed and reported separately, but the results are similar as would be expected for this small change in fat content.

### Exercise Intervention

Mice assigned to the exercise intervention had voluntary access to running wheels. Female C57BL/6 mice readily perform voluntary wheel running up to 7 h nightly and can be compared with sedentary control animals housed in identical conditions without wheel access (17, 18). Exercise wheels are equipped with a Mity 8 Cyclocomputer (model CC-MT400), which records distance, average speed, running time, and maximum speed. Quadriceps muscles were harvested after 6 weeks of running.

### Calorimetry

Indirect calorimetry (TSE Systems) metabolic cages were utilized to evaluate oxygen consumption, carbon dioxide expenditure, and activity by infrared counts of animal movement. Whole-chamber temperature (72°F/22.2°C) and humidity were regulated. Mice were placed in the calorimetry cages for 24 h in order to acclimate to their new surroundings. After 24 h, calorimetry instrumentation was used to evaluate energy expenditure in mice. The resulting respiratory exchange ratios (RERs) and energy expenditure values were calculated from these measurements. Calorimetry testing was performed for 48 h.

### MRI

For the long-term HFD experiment, MRI technology was used to evaluate whole body composition (including fat, lean tissue, and water) *in vivo* 3 weeks after initiation of exercise intervention (15 weeks after initiation of the experiment). Body composition was assessed using MRI (EchoMRI, Houston, TX, USA) to determine fat and lean mass percentages, as described previously (19).

### RNA Isolation from Muscle

Total RNA from quadriceps muscle was isolated and 1 μg was reverse transcribed and analyzed *via* real-time PCR, as described previously (18). The quadriceps muscle was selected due to its known exercise response compared with other muscles in the lower limb (20, 21). Ten microliters of cDNA from each experimental condition were pooled and diluted 1:10 to 1:10,000 to generate a five-point standard curve. A non-template control was added to each PCR reaction. Standards and samples were run in duplicate and PCR products normalized to GAPDH amplicons.

### Triglyceride Assay

Muscle tissue homogenate was assayed for triglyceride content using a commercially available colorimetric assay from Cayman (Cayman #10010303). Manufacturer protocol specifies the use of whole tissue homogenates and, therefore, lipids are not isolated prior to us of this TG assay.

### Western Blotting

Protein was isolated and blotted, as described previously (22). Briefly, muscle homogenates (15–20 μg) were loaded onto a polyacrylamide gel for chromatography and transferred to PVDF membrane. After blocking, primary antibody to UCP1 (U6382 SIGMA) was applied overnight at 4°C. Secondary antibody conjugated with horseradish peroxidase was detected with ECL plus chemiluminescence kit (Amersham Biosciences, Piscataway, NJ, USA).

### Statistical Analysis

Statistical significance was evaluated by unpaired *t*-test (two groups) or two-way ANOVA with correction for multiple comparisons *via* a Tukey *post hoc* test (GraphPad Prism 6.05). Exercise and dietary intervention (HFD) were used as the analysis variables.

## Results

### Effects of Diet and Exercise on Body Weight, Body Composition, and Calorimetry

**Figure 1:**
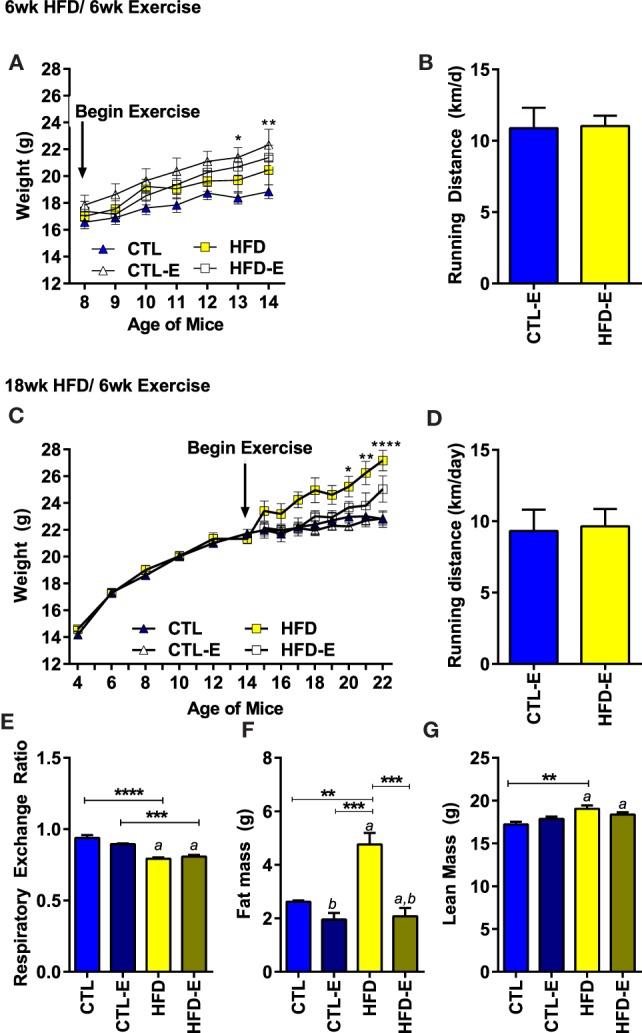
**Body weight and running distance during the short-term and long-term HFD experiments**. **(A)** Weight in grams. **(B)** Average running distance in kilometer per day. **(C)** Weight in grams in long-term HFD experiment. **(D)** Average running distance in kilometer per day in long-term HFD experiment. **(E)** Calorimetry was used to measure the respiratory exchange ratio (RER) in the long-term, 18-week HFD, experiment 3 weeks after initiation of exercise. During calorimetry testing, mice did not have access to running wheels. **(F)** Fat mass via MRI (grams). **(G)** Lean mass via MRI (grams). Results expressed as means ± SEM. Statistical significance designated on graphs as follows: ● = trend (*p*-value <0.10); *p-value <0.05; **p-value <0.01; ***p-value <0.001; ****p-value <0.0001. ^a^Significant for diet effect by two-way ANOVA. ^b^Significant for exercise effect by two-way ANOVA.

Animal weight and running distance are shown in Figure 1. When mice were switched to a HFD along with beginning the running intervention (6-week HFD/6-week exercise), there were no differences in weight gain due to diet or exercise (Figure 1A). By contrast, runners on the CTL diet weighed significantly more than sedentary CTLs after 6 weeks. The type of diet had no effect on the average running distance per day (Figure 1B).

To understand if running could alter the fat content of mice obese at the beginning of the exercise intervention, we performed a long-term diet experiment: mice were begun on HFD 12 weeks prior to separation into exercise and sedentary groups (18-week HFD/6-week exercise). In the long-term HFD experiment, a significant weight difference was achieved between the HFD-fed and CTL mice by the last 3 weeks of the experiment (Figure 1C, Final weight CTL 22.8 ± 1.6, HFD 27.2 ± 2.0, *p* < 0.0001). Perigonadal fat pads weighed more in 18-week HFD (0.84 ± 0.26 g) vs. CTL animals (0.33 ± 0.11 g) (*p* = 0.009). Once again, neither diet nor weight affected the daily distance run (Figure 1D). Additionally, body composition was analyzed using MRI 3 weeks prior to harvest in the long-term experiment. After 3 weeks of running, at which time point MRI and calorimetry testing was performed – animal weights were as follows: CTL (21.53 ± 0.5 g; CTL), CTL-E (21.6 ± 0.7), HFD (25.6 ± 1.4 g), HFD-E (22.2 ± 0.69). Fat mass by MRI was doubled by the 18-week HFD group (4.8 ± 0.4 g) compared with the 18-week HFD-E group 2.1 ± 0.3 g (Figure 1F, *p* < 0.001). Lean mass was higher in the 18-week HFD group relative to CTL (Figure 1G, *p* < 0.01) but was not significantly altered by exercise (Figure 1G).

Three weeks prior to harvest, calorimetry was performed in the long-term HFD experiment to ascertain fuel source. The RER indicated that fat was the principal fuel source in animals fed a HFD (Figure 1E, 0.94 vs. 0.79, *p* < 0.01). Running exercise did not significantly alter RER (Figure 1E); however, metabolic cage testing was performed in the absence of running wheels for a 48-h period, 3 weeks prior to sacrifice. Reversal of BAT-associated thermogenesis can occur rapidly in as little as 60 min (23). Thus, during calorimetry testing, exercise-induced browning effects are likely reversed early in the process. Diets were maintained during calorimetry testing and, thus, dietary effects persist. This limitation may prevent assessment of changes in RER due to running.

### Running Exercise Increases Muscle Lipid and Fat Markers

**Figure 2:**
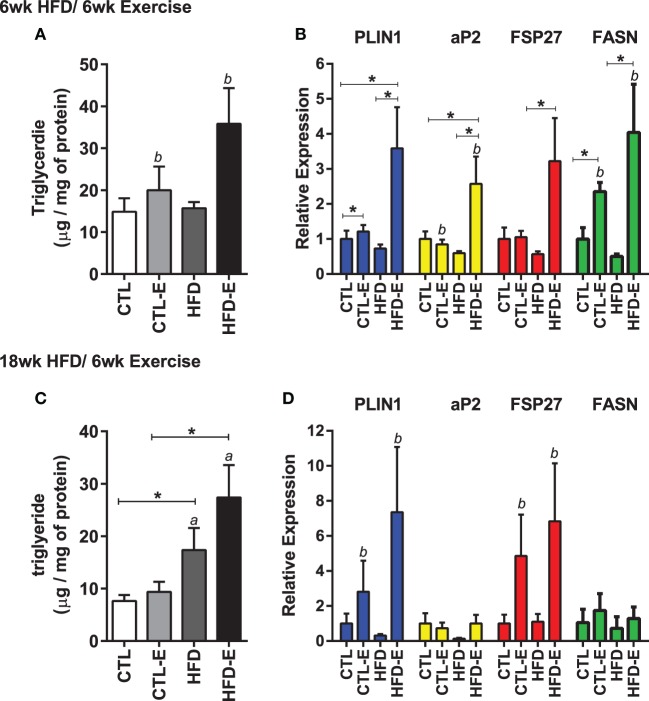
**Running exercise effect on triglyceride and fat formation markers in skeletal muscle**. **(A)** Triglyceride content of quadriceps muscle normalized to mg of protein. **(B)** Skeletal muscle mRNA expression of *FASN, aP2, FSP27*, and *FASN* relative to *GAPDH*. **(C,D)** Triglyceride content and gene expression for 18wk experiment. Results expressed as means ± SEM relative to CTL. Statistical significance designated on graphs as follows: ● = trend (*p*-value <0.10); *p-value <0.05; **p-value <0.01; ***p-value <0.001; ****p-value <0.0001. ^a^Significant for diet effect by two-way ANOVA. ^b^Significant for exercise effect by two-way ANOVA.

In order to understand the effect of running on muscle lipid, we measured triglyceride content in the quadriceps muscle. Muscle TG increased significantly in response to running, but not to diet in the short-term HFD experiment (Figure 2A, *p* < 0.05 for an exercise effect by two-way ANOVA). After 18 week of high fat feeding, muscle TG was substantially increased in HFD groups, accompanying the obesity (Figure 2C, *p* < 0.05 for a diet effect by two-way ANOVA). In the HFD-fed runners, there was a trend for further increases in muscle TG above the non-running cohort (Figure 2C).

We evaluated muscle mRNA expression of fat and lipogenesis markers, including *Plin1, aP2, fat-specific protein 27* (*Fsp27*), and fatty acid synthase (*Fasn*). In the short-term experiment where diet and running measures were concurrently introduced, there was an increase in *Plin1, aP2, Fsp27, and Fasn* in exercisers in CTL and HFD groups (Figure 2B). *Fasn* was 2.3 ± 0.2 in CTL-E vs. 1.0 ± 0.3 in CTL and 4.0 ± 1.4 in HFD-E vs. 0.5 ± 0.1 in HFD (Figure 2B, *p* < 0.05 for an exercise effect by two-way ANOVA). Western blot of aP2 confirmed effects of exercise to increase fat genes in the setting of HFD (Figures 5A,C, *p* < 0.05). Overall, the combination of HFD and exercise caused significant increases in fat markers by 6 weeks (Figure 2A).

In the long-term experiment, where the high-fat diet was delivered for 12 weeks prior to institution of exercise for a subsequent 6 weeks, *FSP27* and *Plin1* were both increased with exercise, in both diet groups (Figure 2D, *p* < 0.05 for an exercise effect by two-way ANOVA). Exercise did not affect the fat markers *aP2* and *Fasn* in the long-term HFD experiment and this differed from the short-term experiment (Figure 2D). These differences may be due to age of mice, or the accrued effects of 18 weeks of HFD.

**Figure 3:**
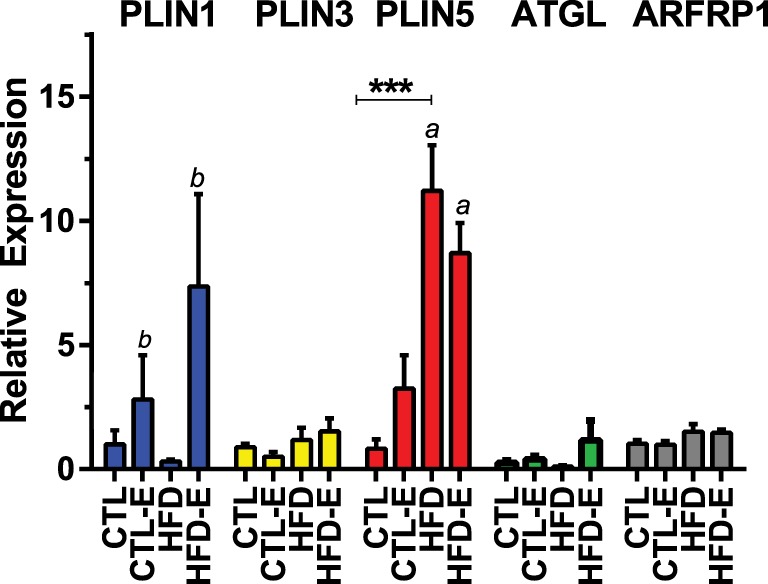
**Effects of diet and exercise on lipid droplet-associated markers**. Skeletal muscle mRNA expression of *ATGL, Plin3, Plin5*, and *ARFRP1* relative to *GAPDH* in the 18-week HFD mice. Results expressed as means ± SEM relative to CTL. Statistical significance designated on graphs as follows: ● = trend (*p*-value <0.10); *p-value <0.05; **p-value <0.01; ***p-value <0.001; ****p-value <0.0001. ^a^Significant for diet effect by two-way ANOVA. ^b^Significant for exercise effect by two-way ANOVA.

We also investigated whether perilipin subtypes were affected by diet and/or exercise, as a recent study suggested that differential expression of perilipin 3 and perilipin 5 might explain differential lipid oxidation efficiency in skeletal muscle in humans (24). Perilipin 1 or *Plin1*, the most abundant of the perilipins (25, 26), rose in response to exercise, more significantly in the HFD-fed runners (Figures 2B,D and 3). *Plin5* was increased more significantly in response to HFD (Figure 3, *p* < 0.0001 for a diet effect by two-way ANOVA). *Plin5* was suggested to rise in muscle of physically active humans compared with sedentary subjects (24); however, in our mice, exercise did not alter *Plin5*. We also evaluated *Plin3* expression as its deletion has been shown to impair lipid oxidation in human myotubes (24): we found no differences in *Plin3* expression in response to diet or exercise in murine muscle. With regard to lipid energetics, the ARF-like GTPase *ARFRP1* is involved in lipid droplet growth and regulation of lipolysis. In our experiment, exercise failed to regulate *ARFRP1* or *ATGL* that is also involved in lipolysis.

### Running Exercise Increases Markers of Brown Adipose Tissue in Skeletal Muscle

**Figure 4:**
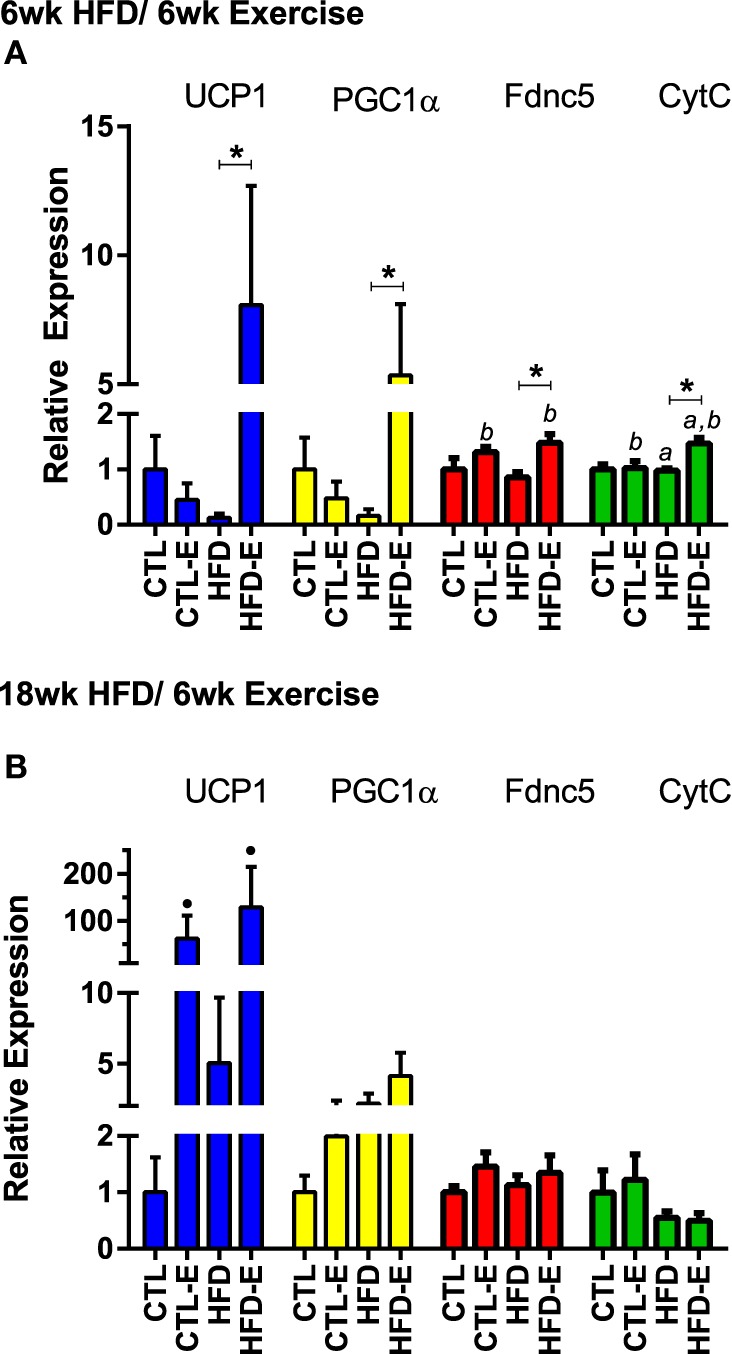
**Running exercise effect on markers of brown adipose tissue in skeletal muscle**. **(A)** Short-term HFD experiment skeletal muscle mRNA expression of *UCP1, PGC1a, Fndc5*, and *CytC* relative to *GAPDH*. **(B)** Results from long-term HFD experiment. Results expressed as means ± SEM relative to CTL. Statistical significance designated on graphs as follows: ● = trend (*p*-value <0.10); *p-value <0.05; **p-value <0.01; ***p-value <0.001; ****p-value <0.0001. ^a^Significant for diet effect by two-way ANOVA. ^b^Significant for exercise effect by two-way ANOVA.

To characterize the muscle lipid, we evaluated muscle mRNA expression of markers associated with brown adipose tissue. These included *Fndc5*, the transcript for irisin, *Pgc1*α (regulator of mitochondrial function), *Ucp1* (uncoupling protein found in the mitochondria of brown adipose tissue), and *CtytC* (component in the electron transport chain of mitochondria). In the short-term experiment, *Fndc5* expression increased with exercise in both CTL and HFD groups (Figure 4A, *p* < 0.05 for exercise effect *via* two-way ANOVA). *Pgc1*α increased in HFD-E by 5.3-fold (Figure 4A, *p* < 0.05 for HFD-E vs. HFD) (Figure 4A). *Ucp1* was increased 8.1-fold in HFD-E and was downregulated in the HFD-control mice by 0.13-fold (Figure 4A, *p* < 0.01 for HFD-E vs. HFD). *CytC* increased significantly due to exercise in both CTL and HFD groups (Figure 4A, *p* < 0.05 for an exercise effect by two-way ANOVA).

**Figure 5:**
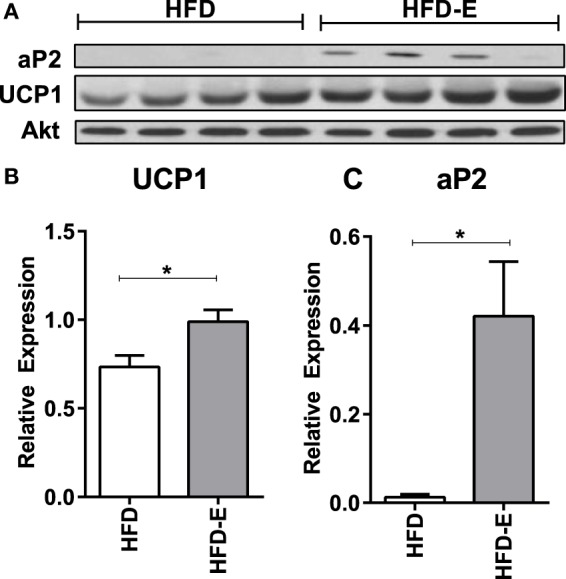
**Exercise increases UCP1 protein expression in skeletal muscle in the setting of high fat feeding**. **(A)** Western blots of skeletal muscle UCP1 and aP2 of HFD-controls and runners in the short-term HFD experiment. **(B)** UCP1. **(C)** aP2. Statistical significance designated on graphs as follows: ● = trend (*p*-value <0.10); *p-value <0.05; **p-value <0.01; ***p-value <0.001; ****p-value <0.0001. ^a^Significant for diet effect by two-way ANOVA. ^b^Significant for exercise effect by two-way ANOVA.

In the long-term experiment, *Ucp1* demonstrated a trend for increase with exercise: CTL 1.0 ± 0.6, CTL-E 62.7 ± 48.7, HFD 5.0 ± 4.6, HFD-E 129.2 ± 86.0 (Figure 4B, *p*-value = 0.09 for exercise effect by two-way ANOVA). Other brown fat markers were unaffected by either exercise or diet. The trend, but lack of a significance for an effect of exercise to induce brown fat markers in older mice may be due to the higher level or persistence of muscle lipid after 18 weeks of HFD. UCP1 rise in response to exercise was confirmed by western blotting in the setting of HFD (Figure 5, *p* < 0.05).

## Discussion

In this study of skeletal muscle, we found that running exercise in mice increased muscle lipid. Exercise induction of muscle lipid increase was most notable in the already increased muscle lipid of mice fed with a high-fat diet. We found that *Fasn*, the transcript for an enzyme that initiates *de novo* lipogenesis, was increased in the setting of exercise. Our findings of increased triglyceride, *Plin1*, and *Fasn* mRNA are consistent with prior studies examining the effects of exercise on lipogenesis in skeletal muscle (27, 28). This work suggests a potential mechanism that could explain the *athlete’s paradox*, although additional investigation is required to confirm these effects in humans. Importantly, we found that phenotypic markers of beige/brown fat, including UCP1 and aP2 increased in muscle of exercisers, suggesting a phenotypic switch to a beige/brown muscle lipid in the setting of exercise.

Prior work has shown increased insulin sensitivity and oxidative capacity in skeletal muscle of trained endurance athletes, as opposed to the skeletal muscle of diabetics, which has lower insulin sensitivity and oxidative capacity (5). During exercise, fatty acids from the hydrolysis of muscle lipid contribute a substantial portion of the energy required for oxidative metabolism (29). Thus, it has been proposed that the increased myocellular TG in the setting of exercise serves as a fuel for the metabolic needs of exercise (5). It has been proposed that pathologic muscle lipid, such as that which accumulates in the setting of obesity or diabetes, contains lipotoxic intermediates, and it is these, rather than the absolute presence of lipid, which mediate insulin resistance (30). Some studies have shown that exercise lowers lipotoxic lipid intermediates diacylglycerol and ceramide (31, 32); however, others did not find an effect of exercise training on diacylglycerol and ceramide levels (33). At this time, there are insufficient data with regard to the exercise effect on lipid intermediates to explain the *athlete’s paradox*. It is notable that we found a differential expression of *Plin5* (increased by HFD) as opposed to *Plin1* (increased by exercise). *Plin5* is a lipid droplet protein that promotes association of lipid droplets with mitochondria and is expressed in oxidative tissues, including cardiac and skeletal muscle (34). *Plin1*, on the other hand, is the most abundant perilipin, which potently suppresses basal lipolysis and is present in both white and brown adipose tissue (26, 34). Some have proposed that exercise may increase turnover of muscle lipid and, therefore, prevent accumulation of lipotoxic intermediate lipid species (35). Lipid uptake has also been analyzed and exercising mice were found to have similar levels of *lpl* mRNA and *CD36* compared with non-exercisers, suggesting that lipid uptake does not play a significant role in the *athlete’s paradox* (28). Moreover, reduced lipolysis does not explain the increased muscle lipid in the setting of exercise as we found *Plin5* as well as *ATGL* to be unaffected by exercise. *Plin5* is the only perilipin to directly bind ATGL Exercise has been shown to prevent lipolysis (36). Based on the metabolic benefits of exercise (37), we propose that exercise-associated muscle lipid might improve muscle function, perhaps providing myocytes with high-quality energy storage that is readily accessible for long distance muscle contraction.

Previous reports have suggested that muscle lipid associated with the *athlete’s paradox* can be augmented by diet composition (38, 39). Endurance runners, for instance, had three times the amount of muscle lipid in the tibialis anterior muscle after eating a high-fat diet vs. a normal fat diet (39). Both a high-fat diet and exercise are known to increase muscle lipid and this rise is likely additive when both are combined (4, 5). Our data support that exercise increases muscle lipid, and that the substitution of fat calories in the diet augments the exercise effect.

In our study of muscle lipid, the most significant change measured due to exercise was the increase in *UCP1*, which supports that the muscle fat became more brown. While there are other uncoupling proteins, there is little to suggest that these have an impact on energy expenditure (40, 41). Almind et al. (42) noted that 129 mice, protected from diet-induced obesity due to differences in energy expenditure, showed higher UCP1 expression in mitochondria of brown adipocytes found within muscle. Thus, the browning of an ectopic fat depot appears to protect from obesity.

Increased expression of *PGC1*α in skeletal muscle has been shown to stimulate the expression of *Fndc5*, encoding for irisin (12). Irisin, considered to be a humoral factor from muscle that stimulates browning of fat in white adipose tissue, at least in mice (12). Our results suggest that exercise-induced browning, perhaps even dependent on PGC1a stimulated irisin, is recapitulated within the muscle lipid of our running mice: we measured small but significant increase in levels of the irisin transcript in CTL-E and HFD-E in our short-term HFD experiment.

Since brown adipose tissue and skeletal muscle share developmental origins and brown adipocyte progenitors have been noted in human skeletal muscle (43, 44), it is plausible that the muscle fat could be browned. Low levels of *UCP1* mRNA have been identified in human muscle biopsies, also hinting at the presence of brown adipocytes that potentially arise from myocyte precursors (11, 43). Alternatively, UCP1 may represent a novel expression of this metabolic regulator in skeletal muscle, a site that has been demonstrated to significantly contribute to adaptive thermogenesis (45). Our study showed an increase in markers consistent with the brown adipose phenotype in muscle of running mice concurrently fed a HFD. Additionally, *Plin1*, increased in muscle of runners in our experiment, has been shown to regulate thermogenesis in brown adipose tissue (46). This suggests that dietary-induced muscle lipid can be altered by exercise to have different consequences than white phenotypic lipid stores. Interestingly, in one study where obese and sedentary humans were started on a mild exercise regimen, examination of muscle showed that the muscle lipid, while unchanged in amount, was dispersed into smaller droplets (47). As this change in lipid droplet size was associated with an increase in oxidative capacity and insulin sensitivity (47), this may suggest that even mild exercise can alter the phenotype of muscle lipid.

The main limitation of this study is the inability to distinguish between intramyocellular and extramyocellular lipid; this distinction will be important for future studies. Lipid localization with histology, staining for UCP1 and magnetic resonance imaging is planned for future studies as well. Additionally, calorimetry cages do not contain running wheels and, therefore, calorimetry testing was solely able to assess the effects of diet – as opposed to exercise – on the RER.

In conclusion, this work puts forth a potential mechanism that could explain the *athlete’s paradox*, although additional investigation is required to confirm these effects in human clinical trials. Additionally, HFD was additive to exercise in browning muscle lipid and, thus, exogenous lipid consumption may be a critical factor in the phenotypic shifts that occur with endurance training. This mouse model suggests that increased muscle lipid may represent a potential beige fat depot that serves the metabolic needs of exercise.

## Author Contributions

TM carried out experimental design, experiments, data collection, data analysis, and manuscript preparation. KG carried out experiments, data collection, data analysis, and manuscript revision. CM carried out experiments, data collection, data analysis, and manuscript revision. XW carried out experiments, data collection, data analysis, and manuscript revision. GU carried out data analysis and manuscript revision. GBU carried out experiments and data collection. BS carried out data analysis and manuscript revision. ZX carried out manuscript revision. DT carried out experiments, data collection, data analysis, and manuscript revision. JR carried out experimental design and manuscript revision. MS carried out experimental design, performed experiments, data collection, data analysis, and manuscript preparation.

## Conflict of Interest Statement

The authors declare that the research was conducted in the absence of any commercial or financial relationships that could be construed as a potential conflict of interest.

## References

[1] Pan DA Lillioja S Milner MR Kriketos AD Baur LA Bogardus C Skeletal muscle membrane lipid composition is related to adiposity and insulin action. J Clin Invest (1995) 96(6):2802–8. 10.1172/JCI118350 8675650 PMC185990

[2] Jacob S Machann J Rett K Brechtel K Volk A Renn W Association of increased intramyocellular lipid content with insulin resistance in lean nondiabetic offspring of type 2 diabetic subjects. Diabetes (1999) 48(5):1113–9. 10.2337/diabetes.48.5.1113 10331418

[3] Goodpaster BH Theriault R Watkins SC Kelley DE. Intramuscular lipid content is increased in obesity and decreased by weight loss. Metabolism (2000) 49(4):467–72. 10.1016/S0026-0495(00)80010-4 10778870

[4] Bachmann OP Dahl DB Brechtel K Machann J Haap M Maier T Effects of intravenous and dietary lipid challenge on intramyocellular lipid content and the relation with insulin sensitivity in humans. Diabetes (2001) 50(11):2579–84. 10.2337/diabetes.50.11.2579 11679437

[5] Goodpaster BH He J Watkins S Kelley DE. Skeletal muscle lipid content and insulin resistance: evidence for a paradox in endurance-trained athletes. J Clin Endocrinol Metab (2001) 86(12):5755–61. 10.1210/jcem.86.12.8075 11739435

[6] van Loon LJ Koopman R Manders R van der Weegen W van Kranenburg GP Keizer HA. Intramyocellular lipid content in type 2 diabetes patients compared with overweight sedentary men and highly trained endurance athletes. Am J Physiol Endocrinol Metab (2004) 287(3):E558–65. 10.1152/ajpendo.00464.2003 15165998

[7] Kiens B. Skeletal muscle lipid metabolism in exercise and insulin resistance. Physiol Rev (2006) 86(1):205–43. 10.1152/physrev.00023.2004 16371598

[8] Hawley JA Hargreaves M Joyner MJ Zierath JR Integrative biology of exercise. Cell (2014) 159(4):738–49. 10.1016/j.cell.2014.10.029 25417152

[9] Cannon B Nedergaard J. Brown adipose tissue: function and physiological significance. Physiol Rev (2004) 84(1):277–359. 10.1152/physrev.00015.2003 14715917

[10] Nedergaard J Bengtsson T Cannon B. Unexpected evidence for active brown adipose tissue in adult humans. Am J Physiol Endocrinol Metab (2007) 293(2):E444–52. 10.1152/ajpendo.00691.2006 17473055

[11] Wu J Bostrom P Sparks LM Ye L Choi JH Giang AH Beige adipocytes are a distinct type of thermogenic fat cell in mouse and human. Cell (2012) 150(2):366–76. 10.1016/j.cell.2012.05.016 22796012 PMC3402601

[12] Bostrom P Wu J Jedrychowski MP Korde A Ye L Lo JC A PGC1-alpha-dependent myokine that drives brown-fat-like development of white fat and thermogenesis. Nature (2012) 481(7382):463–8. 10.1038/nature10777 22237023 PMC3522098

[13] Szczepaniak LS Babcock EE Schick F Dobbins RL Garg A Burns DK Measurement of intracellular triglyceride stores by H spectroscopy: validation in vivo. Am J Physiol (1999) 276(5 Pt 1):E977–89. 10329993 10.1152/ajpendo.1999.276.5.E977

[14] Schrauwen-Hinderling VB Kooi ME Hesselink MK Moonen-Kornips E Schaart G Mustard KJ Intramyocellular lipid content and molecular adaptations in response to a 1-week high-fat diet. Obes Res (2005) 13(12):2088–94. 10.1038/oby.2005.259 16421342

[15] Allen DL Harrison BC Maass A Bell ML Byrnes WC Leinwand LA. Cardiac and skeletal muscle adaptations to voluntary wheel running in the mouse. J Appl Physiol (2001) 90(5):1900–8. 11299284 10.1152/jappl.2001.90.5.1900

[16] Styner M Thompson WR Galior K Uzer G Wu X Kadari S Bone marrow fat accumulation accelerated by high fat diet is suppressed by exercise. Bone (2014) 64C:39–46. 10.1016/j.bone.2014.03.044 24709686 PMC4041820

[17] Styner M Pagnotti GM Galior K Wu X Thompson WR Uzer G Exercise regulation of marrow fat in the setting of PPARgamma agonist treatment in female C57BL/6 mice. Endocrinology (2015) 156(8):2753–61. 10.1210/en.2015-1213 26052898 PMC4511140

[18] Styner M Meyer MB Galior K Case N Xie Z Sen B Mechanical strain downregulates C/EBPbeta in MSC and decreases endoplasmic reticulum stress. PLoS One (2012) 7(12):e51613 10.1371/journal.pone.0051613 23251594 PMC3520924

[19] Ellis JM Li LO Wu PC Koves TR Ilkayeva O Stevens RD Adipose acyl-CoA synthetase-1 directs fatty acids toward beta-oxidation and is required for cold thermogenesis. Cell Metab (2010) 12(1):53–64. 10.1016/j.cmet.2010.05.012 20620995 PMC2910420

[20] Savage KJ McPherron AC. Endurance exercise training in myostatin null mice. Muscle Nerve (2010) 42(3):355–62. 10.1002/mus.21688 20544938 PMC2976492

[21] Gordon BS Lowe DA Kostek MC. Exercise increases utrophin protein expression in the mdx mouse model of Duchenne muscular dystrophy. Muscle Nerve (2014) 49(6):915–8. 10.1002/mus.24151 24375286

[22] Sen B Xie Z Uzer G Thompson WR Styner M Wu X Intranuclear actin regulates osteogenesis. Stem Cells (2015) 33:3065–76. 10.1002/stem.2090 26140478 PMC4788101

[23] Blessing WW Zilm A Ootsuka Y. Clozapine reverses increased brown adipose tissue thermogenesis induced by 3,4-methylenedioxymethamphetamine and by cold exposure in conscious rats. Neuroscience (2006) 141(4):2067–73. 10.1016/j.neuroscience.2006.05.050 16814930

[24] Covington JD Noland RC Hebert RC Masinter BS Smith SR Rustan AC Perilipin 3 differentially regulates skeletal muscle lipid oxidation in active, sedentary, and type 2 diabetic males. J Clin Endocrinol Metab (2015) 100(10):3683–92. 10.1210/JC.2014-4125 26171795 PMC4596049

[25] Nishiu J Tanaka T Nakamura Y. Isolation and chromosomal mapping of the human homolog of perilipin (PLIN), a rat adipose tissue-specific gene, by differential display method. Genomics (1998) 48(2):254–7. 10.1006/geno.1997.5179 9521880

[26] Patel S Yang W Kozusko K Saudek V Savage DB. Perilipins 2 and 3 lack a carboxy-terminal domain present in perilipin 1 involved in sequestering ABHD5 and suppressing basal lipolysis. Proc Natl Acad Sci U S A (2014) 111(25):9163–8. 10.1073/pnas.1318791111 24927580 PMC4078844

[27] Summermatter S Baum O Santos G Hoppeler H Handschin C. Peroxisome proliferator-activated receptor {gamma} coactivator 1{alpha} (PGC-1{alpha}) promotes skeletal muscle lipid refueling in vivo by activating de novo lipogenesis and the pentose phosphate pathway. J Biol Chem (2010) 285(43):32793–800. 10.1074/jbc.M110.145995 20716531 PMC2963391

[28] Summermatter S Shui G Maag D Santos G Wenk MR Handschin C PGC-1alpha improves glucose homeostasis in skeletal muscle in an activity-dependent manner. Diabetes (2013) 62(1):85–95. 10.2337/db12-0291 23086035 PMC3526021

[29] Hurley BF Nemeth PM Martin WH III Hagberg JM Dalsky GP Holloszy JO Muscle triglyceride utilization during exercise: effect of training. J Appl Physiol (1985) (1986) 60(2):562–7. 3512511 10.1152/jappl.1986.60.2.562

[30] Bosma M Kersten S Hesselink MK Schrauwen P. Re-evaluating lipotoxic triggers in skeletal muscle: relating intramyocellular lipid metabolism to insulin sensitivity. Prog Lipid Res (2012) 51(1):36–49. 10.1016/j.plipres.2011.11.003 22120643

[31] Bruce CR Thrush AB Mertz VA Bezaire V Chabowski A Heigenhauser GJ Endurance training in obese humans improves glucose tolerance and mitochondrial fatty acid oxidation and alters muscle lipid content. Am J Physiol Endocrinol Metab (2006) 291(1):E99–107. 10.1152/ajpendo.00587.2005 16464906

[32] Dube JJ Amati F Stefanovic-Racic M Toledo FG Sauers SE Goodpaster BH. Exercise-induced alterations in intramyocellular lipids and insulin resistance: the athlete’s paradox revisited. Am J Physiol Endocrinol Metab (2008) 294(5):E882–8. 10.1152/ajpendo.00769.2007 18319352 PMC3804891

[33] Devries MC Samjoo IA Hamadeh MJ McCready C Raha S Watt MJ Endurance training modulates intramyocellular lipid compartmentalization and morphology in skeletal muscle of lean and obese women. J Clin Endocrinol Metab (2013) 98(12):4852–62. 10.1210/jc.2013-2044 24081737

[34] Kimmel AR Sztalryd C. Perilipin 5, a lipid droplet protein adapted to mitochondrial energy utilization. Curr Opin Lipidol (2014) 25(2):110–7. 10.1097/MOL.0000000000000057 24535284 PMC4517968

[35] van Loon LJ Goodpaster BH. Increased intramuscular lipid storage in the insulin-resistant and endurance-trained state. Pflugers Arch (2006) 451(5):606–16. 10.1007/s00424-005-1509-0 16155759

[36] Wang H Bell M Sreenivasan U Hu H Liu J Dalen K Unique regulation of adipose triglyceride lipase (ATGL) by perilipin 5, a lipid droplet-associated protein. J Biol Chem (2011) 286(18):15707–15. 10.1074/jbc.M110.207779 21393244 PMC3091179

[37] Hu FB Willett WC Li T Stampfer MJ Colditz GA Manson JE. Adiposity as compared with physical activity in predicting mortality among women. N Engl J Med (2004) 351(26):2694–703. 10.1056/NEJMoa042135 15616204

[38] Tamura Y Watada H Igarashi Y Nomiyama T Onishi T Takahashi K Short-term effects of dietary fat on intramyocellular lipid in sprinters and endurance runners. Metabolism (2008) 57(3):373–9. 10.1016/j.metabol.2007.10.013 18249210

[39] de Queiroz KB Rodovalho GV Guimaraes JB de Lima DC Coimbra CC Evangelista EA Endurance training blocks uncoupling protein 1 up-regulation in brown adipose tissue while increasing uncoupling protein 3 in the muscle tissue of rats fed with a high-sugar diet. Nutr Res (2012) 32(9):709–17. 10.1016/j.nutres.2012.06.020 23084644

[40] Fink BD Hong YS Mathahs MM Scholz TD Dillon JS Sivitz WI. UCP2-dependent proton leak in isolated mammalian mitochondria. J Biol Chem (2002) 277(6):3918–25. 10.1074/jbc.M107955200 11723122

[41] Erlanson-Albertsson C. The role of uncoupling proteins in the regulation of metabolism. Acta Physiol Scand (2003) 178(4):405–12. 10.1046/j.1365-201X.2003.01159.x 12864746

[42] Almind K Manieri M Sivitz WI Cinti S Kahn CR. Ectopic brown adipose tissue in muscle provides a mechanism for differences in risk of metabolic syndrome in mice. Proc Natl Acad Sci U S A (2007) 104(7):2366–71. 10.1073/pnas.0610416104 17283342 PMC1892979

[43] Crisan M Casteilla L Lehr L Carmona M Paoloni-Giacobino A Yap S A reservoir of brown adipocyte progenitors in human skeletal muscle. Stem Cells (2008) 26(9):2425–33. 10.1634/stemcells.2008-0325 18617684

[44] Schulz TJ Huang TL Tran TT Zhang H Townsend KL Shadrach JL Identification of inducible brown adipocyte progenitors residing in skeletal muscle and white fat. Proc Natl Acad Sci U S A (2011) 108(1):143–8. 10.1073/pnas.1010929108 21173238 PMC3017184

[45] van den Berg SA van Marken Lichtenbelt W Willems van Dijk K Schrauwen P. Skeletal muscle mitochondrial uncoupling, adaptive thermogenesis and energy expenditure. Curr Opin Clin Nutr Metab Care (2011) 14(3):243–9. 10.1097/MCO.0b013e3283455d7a 21415733

[46] Souza SC Christoffolete MA Ribeiro MO Miyoshi H Strissel KJ Stancheva ZS Perilipin regulates the thermogenic actions of norepinephrine in brown adipose tissue. J Lipid Res (2007) 48(6):1273–9. 10.1194/jlr.M700047-JLR200 17401109

[47] He J Goodpaster BH Kelley DE. Effects of weight loss and physical activity on muscle lipid content and droplet size. Obes Res (2004) 12(5):761–9. 10.1038/oby.2004.92 15166296

