# Supplementary material for: Exercise Increases and Browns Muscle Lipid in High-Fat Diet-Fed Mice
Source: Front Endocrinol (Lausanne). 2016 Jun 30;7:80. doi: 10.3389/fendo.2016.00080 (PMC4928595; doi:10.3389/fendo.2016.00080)
Supplement: Supplementary file 1 [file Table_1.PDF]

Gene	Forward Primer	Reverse Primer	NCBI Reference Sequence
Perilipin 1	5'-CCATGTCCCTATCCGATGCC-3'	5'-TGGGAAGCGGCACATAGTGTA-3'	NM_001113471.1
Perilipin 3	5'-ATGAACACTCCCTCGGCAAG-3'	5'- ATTCCATCAGGCCGAGCAC-3'	NM_025836.3
Perilipin 5	5'-TCTGATTCTCTGACCGCTGC-3'	5'- ACCGGACATTCTGCTGTGTG-3'	NM_025874.3
aP2	5'-GGATTTCCTCACCATCCGGT-3'	5'-TTCACCTTCCTGTCGTCTGC-3'	NM_024406.2
Fsp27	5'-ATGGTGCCAGAGTGGTTAGC-3'	5'-AGAGTCCCAGGTGAGAGACC-3'	NM_178373.3
Fasn	5’-CTACCCAAGACAGGAACCGT-3’	5’-GAGTTCGGGTCTTCCCACAG-3’	NM_007988.3
ARFRP1	5'-CCAAGATCTGAGCAGACTTTCC-3'	5'-ATGAGACGAGCCTTTCCCAC-3'	NM_001165995.1
ATGL	5'-GCATCTCCCTGACTCGTGTT-3'	5'- TGCAGACATTGGCCTGGATG-3'	NM_025802.3
Fndc5	5'-CACGCGAGGCTGAAAAGATG-3'	5'-AGCTATAACACCTGCCCACA-3'	NM_027402.3
Pgc1α	5'-CCCTGCCATTGTTAAGACC-3'	5'-TGCTGCTGTTCCTGTTTTC-3'	NR_027710.1
UCP1	5'-TGGAAAGGGACGACCCCTAAT-3'	5'-ACAGTAAATGGCAGGGGACG-3'	NM_009463.3
CtytC	5’-GCAAGCATAAGACTGGACCAAA-3’	5’-TTCTTGGCATCTGTGTAAGAGAATC-3’	X01756.1

Supplementary table 1. Primer sequences
