# Supplementary material for: Exercise Increases and Browns Muscle Lipid in High-Fat Diet-Fed Mice
Source: Front Endocrinol (Lausanne). 2016 Jun 30;7:80. doi: 10.3389/fendo.2016.00080 (PMC4928595; doi:10.3389/fendo.2016.00080)
Supplement: Supplementary file 2 [file Table_2.PDF]

Peptide/protein target	Antigen sequence (if known)	Name of AB	Manufacturer	Species raised in; mono or polyclonal	Dilution used
aP2		AP-2a AB	Cell Signaling	Rabbit, polyclonal	1:4000
UCP1		UCP1 Rabbit mAB	Cell Signaling	Rabbit, monoclonal	1:1000

Supplementary table 2. Antibodies.
